# When two is worse than one: The deleterious impact of multisensory stimulation on response inhibition

**DOI:** 10.1371/journal.pone.0251739

**Published:** 2021-05-20

**Authors:** Kuzma Strelnikov, Mario Hervault, Lidwine Laurent, Pascal Barone

**Affiliations:** 1 Brain & Cognition Research Center (CerCo), University of Toulouse 3 –CNRS, Toulouse, France; 2 Purpan University Hospital, Toulouse, France; Waseda University, JAPAN

## Abstract

Multisensory facilitation is known to improve the perceptual performances and reaction times of participants in a wide range of tasks, from detection and discrimination to memorization. We asked whether a multimodal signal can similarly improve action inhibition using the stop–signal paradigm. Indeed, consistent with a crossmodal redundant signal effect that relies on multisensory neuronal integration, the threshold for initiating behavioral responses is known for being reached faster with multisensory stimuli. To evaluate whether this phenomenon also occurs for inhibition, we compared stop signals in unimodal (human faces or voices) versus audiovisual modalities in natural or degraded conditions. In contrast to the expected multisensory facilitation, we observed poorer inhibition efficiency in the audiovisual modality compared with the visual and auditory modalities. This result was corroborated by both response probabilities and stop–signal reaction times. The visual modality (faces) was the most effective. This is the first demonstration of an audiovisual impairment in the domain of perception and action. It suggests that when individuals are engaged in a high–level decisional conflict, bimodal stimulation is not processed as a simple multisensory object improving the performance but is perceived as concurrent visual and auditory information. This absence of unity increases task demand and thus impairs the ability to revise the response.

## Introduction

Auditory perception and visual perception have been studied as separate channels, even though the ecological environment is rarely unimodal, and auditory and visual stimuli often combine to provide complementary information about a single multimodal object. There is now a large body of literature clearly demonstrating that integrating sensory information from different channels leads, at both perceptual and neuronal levels, to a different kind of processing from that for a single modality. At the behavioral and perceptual levels, *multisensory integration* reduces perceptual ambiguity, lowers sensory thresholds, and enhances the speed and accuracy of stimulus detection [1, 2]. The perceptual benefit of multisensory integration is supported by direct functional interactions between sensory areas of different modalities, from the early stages of sensory processing to more integrative levels [3]. Across species, behavioral facilitation by multisensory stimuli manifests itself in shorter reaction times (RTs), compared with unimodal RTs in what is known as the *redundant signals effect* [4]. RTs for audiovisual stimulation often violate race models between sensory modalities, which predicts that the faster of the two stimuli in competition mediates the behavioral response in any given trial [5–7]. According to the co–activation model [8], which relies on multisensory neuronal integration, the threshold for initiating behavioral responses is reached faster with multisensory stimuli. The fact that this multisensory gain exceeds the facilitation predicted by summing separate response probabilities points to a form of neuronal integration, rather than simply an accumulation of sensory information [9].

Research on audiovisual integration has clearly revealed the greater efficiency of congruent audiovisual stimulation relative to unimodal stimuli, in terms of both RTs and response probabilities [10]. Most of these studies were designed to elicit a response action, such as pressing a response button in a detection, discrimination, or memorization task [11]. However, there is often a need to stop action in response to a certain signal in ecological and social situations. One quite standard approach to testing the capacity of a signal to inhibit action is the *stop–signal paradigm* [12], in which participants need to respond quickly to a specific main stimulus, but sometimes that main stimulus is followed by a stop signal, upon which participants are supposed to cancel their response. According to the horse–race model [12, 13], successful inhibition in stop–signal tasks relies on the outcome of a race between independent go and stop processes. Inhibitory control fails when the go process finishes the race before the stop one and succeeds when the stop process reaches the response threshold first. The statistical model underlying the horse–race model provides a means of estimating the duration of the covert action inhibition process, in the form of a stop–signal reaction time (SSRT). In this paradigm, inhibitory control can thus be described by both the inhibition function (i.e., probability of responding as a function of the time that elapses between the main stimulus and the stop signal) and the SSRT [14]. The ability to inhibit action is a central executive function that is crucial in various situations and plays a role in several psychopathologies [15].

However, the importance of sensory processes in inhibitory mechanisms has only recently attracted the attention of researchers. Several differences in efficiency between auditory and visual stop–signals have been reported, albeit with non–ecological stimuli [16]. In addition, authors recently found that manipulating the sensory properties of the stimuli influenced inhibitory performance [17]. This influence was associated with activation differences in the cortical network known to underlie inhibitory control, highlighting the interactions between low–level sensory content and high–level control of action processes [17, 18]. Importantly, a redundant signal effect has been found to improve the inhibitory performances when presenting two congruent visual stop signals as compared to the use of a single visual stop signal [19]. Still, such redundant signal effect has not been investigated with bimodal or ecological stimulations, and it remains unknown whether audiovisual integration is beneficial not only for initiating action, but also for inhibiting one. Indeed, providing that a multimodal stop signal leads to a redundant signal effect, the stop process’s resulting shortening would, in the context of the stop–signal paradigm, generate lower response probability and shorter SSRT than unimodal stop signals. Such a hypothesis is based on the widely accepted model of a convergence of the different modalities leading to an integration mechanism that occurs at the low level of information processing in early sensory areas. Numerous studies have described a shortening of sensory responses of single cells responses [20] and an influence on motor processing speed [21]. Indeed, a recent study has demonstrated that multisensory integration accelerates neural processing at the sensory encoding stages as well as during decision formation [22]. In contrast, poorer inhibitory performances obtained with a multimodal stop signal, compared to unimodal ones would indicate either a failure of the a multisensory integration in the stop–signal processing or a strong regulation of multisensory integration through top–down control [23]. Such absence of multisensory benefits would suggest that response inhibition mechanisms could be segregated from those of action, adding further evidence that the cortical mechanisms of multisensory integration are clearly dependent on the behavioral goal.

In the present study, we compared the efficiency of auditory, visual, and audiovisual stop signals using ecological communication stimuli. We hypothesized that as multisensory integration leads to shorter RTs, the same should apply in a stop–signal paradigm, with an audiovisual stop signal leading to shorter SSRTs and lower response probabilities than a unimodal one. We tested our results against the independent race model to ensure that SSRTs were correctly simulated. In addition, we assessed the impact of ecological semantic load on action inhibition. The semantic content of stimuli, especially human faces, is known to enhance accuracy and RTs in detection and categorization tasks [24]. Similarly, the human voice is considered to be a specific communication signal [25], and vocal stimuli, like faces, are processed in specific cortical regions [26]. To test the semantic load effect, we used both natural and degraded stimuli in a classic stop–signal paradigm.

## Materials and methods

### Participants

Participants were 30 healthy volunteers (see below): 10 (six women) participated in the visual experiment; 10 (five women) in the auditory experiment; and 10 (five women) in the audiovisual experiment. Participants were aged 19–30 years (*M* = 23 years). They had normal or corrected–to–normal vision, and no hearing problems were reported. Participants provided sociodemographic characteristics and written informed consent. The study was conducted according to the principles stated in the Declaration of Helsinki (2013) and was approved by the local research ethics committee (Comité Consultatif de Protection des Personnes dans la Recherche Biomédicale Toulouse II Avis N°2–03–34/Avis N°2).

### Apparatus and stimuli

Visual stimuli were displayed on a computer screen (19’’) with a refresh rate of 91 Hz. The computer was equipped with the Serial Response Box™ (SRB), which featured five buttons. One button was used to record the RT. The SRB was placed on the desk in front of the computer. The stimuli were presented using E–Prime software.

In the main task, the visual stimuli were static black–and–white images of different categories (animals, objects, nature scenes [27], except for faces). The images were normalized for intensity, luminance, and contrast. The same set of images was used as visual (only) stimuli for the three experiments. The only difference between groups was the stop–signal modality used after the main task visual stimulus.

In the visual modality, the stop signal was a static neutral female face in the ecological condition and a degraded face (using Fourier phase randomization) in the other condition. In the auditory modality, the stop signal was a female voice saying "Bah" in the ecological condition. In the other condition, the same vocal stimulus was degraded by the 2–band vocoder to create the stop signal [28]. In the audiovisual modality, the stop signal in the ecological condition was a combination of the static neutral female face and the female voice saying "Bah", and in the other condition, the face and the “Bah” sound were both degraded, as described above. A static image was used under the assumption that using a dynamic face would be crucial only for the realization of tasks of recognition of emotions present on faces [29]. The stimuli onsets were closely aligned with a precision of 5 ms.

The stimuli used as stop signals were similar to the ones we used in previous studies based on the same detection task with the same protocols of RTs evaluation [30, 31]. When presented in a visuo–auditory modality, this type of signals induces a shortening of the RTs resulting from a multisensory integration as revealed by the violation of the race model. By accepting a Type 1 risk *α* of .05 and a statistical power 1 –*β* of .95 and an effect size .25 (number of groups 3, number of measures 8, correlations of repeated measures 0.5, non–sphericity correction 1) we can estimate the number of participants for the “ANOVA repeated measures within–between interactions” (using G*power 3.1.9.7 software [32]) as 30 participants, i.e., 10 participants per group. Moreover, in previous studies, SSRT values were convincingly compared between conditions when manipulating stimulation modality in 10 participants [33] or response modality in 9 participants [34].

### Procedure

In each trial, a white fixation cross was followed by the main task image, for which participants had to press the response button as quickly as possible. In 20% of the trials (stop trials), the main task stimulus was followed by the stop signal requiring the participant to cancel the response. Presentation of the fixation cross varied between 750 and 1500 ms, to prevent automatic reactions. The main task stimulus remained on the screen for 80 ms. Stimulus onset asynchrony (SOA) between the end of the main task stimulus and the stop signal varied randomly (0 ms, 25 ms, 50 ms, 75 ms, or 100 ms). Participants were asked to focus on the main task image requiring quick responses. They were also instructed that in some stop trials, they would fail to cancel the response but that they should not be troubled by these failures.

Participants in each group (auditory, visual, and audiovisual) performed 200 trials in each condition (ecological vs. degraded stop signals). The session was divided into four experimental blocks (100 trials per block).

Results on response probabilities were set against the independent race model for stop signal tasks developed by Boucher et al. [13] (see S1 File), and SSRTs were calculated using the integration method devised by Verbruggen and Logan [35]. Briefly, for each SOA, the main stimulus RTs for no–stop trials were rank–ordered, and the *n*^*th*^ RT was selected, where *n* was the number of RTs multiplied by *p*(response_(SOA)_). The SOA was then subtracted to estimate the SSRT. Estimated SSRTs for different SOAs were then averaged to obtain a single SSRT for each participant, by condition.

## Results

### Effects of stimulation modality

#### Response probabilities

We began by assessing the efficiency of each sensory modality in terms of response probability in the stop–signal task. Given that the distribution of response probabilities differed significantly from normal (Kolmogorov–Smirnov test; *p* = .021), we used nonparametric statistics to assess the effect of stimulation modality on the probability of responding.

**Fig 1:**
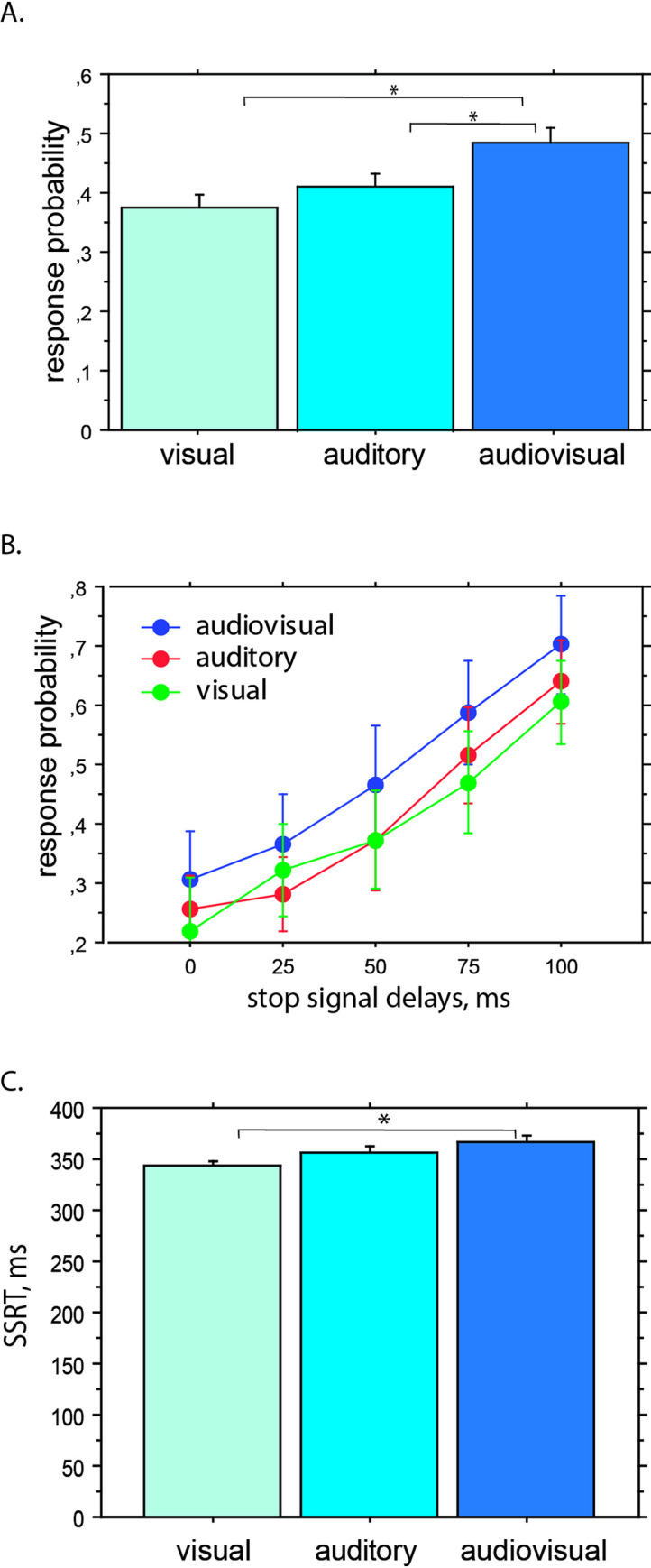
Effect of modality on response probability and stop–signal reaction time. *Note*. Panel A indicates the effect of stimulation modality on response probability after the stop signal. Response probability was significantly lower (meaning more efficient inhibition) for the visual and auditory stop signals than for the audiovisual stop signals. Panel B illustrates the changes in response probability as a function of SOA duration. Across all modalities, response probability increased with the stop signal delay. Panel C shows the effects of stimulation modality on SSRTs. SSRTs were significantly longer for audiovisual stop signals than for visual ones. * *p* < .05.

The effect of stop signal modality, as reflected by response probability, could be assessed either overall or with respect to the various SOAs between the main task stimulus and the stop signal. For the former, the Kruskal–Wallis test showed a significant effect of modality on response probability (H = 7.78, *p* = .021, df = 2), which was significantly higher in the audiovisual modality than in either the auditory (Mann–Whitney test, U = 4087.5, z = –2.23, *p* = .025, *f* = .41) or visual (Mann–Whitney test, U = 3959.5, z = –2.54, *p* = .011, *f* = 0.40; Fig 1A) modalities. The multisensory stop signal was, therefore, statistically less efficient than the unimodal ones. No significant difference in response probability was found between the visual and auditory modalities of the stop signal (Mann–Whitney test, U = 4964.5, z = –1.23, *p* = .223).

Concerning the analysis of the different SOAs, as classically reported in stop–signal experiments [14], we observed that the probability of canceling an action decreased when the interval between the presentation of the main task stimulus and the stop signal increased. Response probability significantly depended on the stop signal delay (Kruskal–Wallis test, *p* = .0001; Fig 1B), insofar as it was easier to cancel the response for short SOAs. This was observed in the visual (Kruskal–Wallis test H = 35.08, *p* = .0017), auditory (Kruskal–Wallis test,H = 47.49, *p* = .0001), and audiovisual (Kruskal–Wallis test, H = 36.97, *p* = .0007) modalities.

#### Stop–signal reaction times

We verified that the main task stimulus RTs for the failed stop trials were shorter on average than RTs for the no–stop trials (*t*–test, *p* = .034), thus validating the race model to compute SSRTs with our data [13, 14]. See also S1 File for the correspondence of our results with the race model of Boucher et al. [13]. As SSRT distribution did not differ significantly from normal (Kolmogorov–Smirnov test; *p* = .22), we performed an analysis of variance (ANOVA) with original stimulation modality as a factor and observed a significant effect of modality on SSRT, *F*(2, 147) = 3.5, *p* = .032. A post hoc Fisher comparison revealed that the mean SSRT was longer for the audiovisual modality (*M* = 367 ms) than for the visual modality (*M* = 344 ms), (*p* = .009, *d* = .38; Fig 1C). This difference reinforced the results on response probability and demonstrated that audiovisual stop signals were less efficient than visual or auditory ones. In order to further validate that this difference was imputable to inhibitory processes, we compared RTs to the main task image between the visual (*M* = 428 ms) and audiovisual (*M* = 430 ms) modalities. No difference was found (Fisher test, *p* = .42, *d =* .*02*), indicating that participants were similarly engaged in the main task, independently of the group modality.

### Effects of stimulation degradation

#### Response probabilities

We further explored the impact of semantic content on the efficiency of the stop signal by comparing the original signals with their degraded counterparts. When we distinguished between the two conditions, we observed an effect of sensory modality on response probabilities with the original stop signals (Kruskal–Wallis test, H = 6.31, *p* = .043, df = 2). In particular, the original audiovisual stop signal had a higher response probability than the visual modality (*p* = .0014), but the difference between the original audiovisual and auditory modalities only tended toward significance (U = 1013.5, z = –1.63, *p* = .10). We failed to find an effect of sensory modality with the degraded stop signals (Kruskal–Wallis test; H = 2.59, *p* = .275, df = 2).

**Fig 2:**
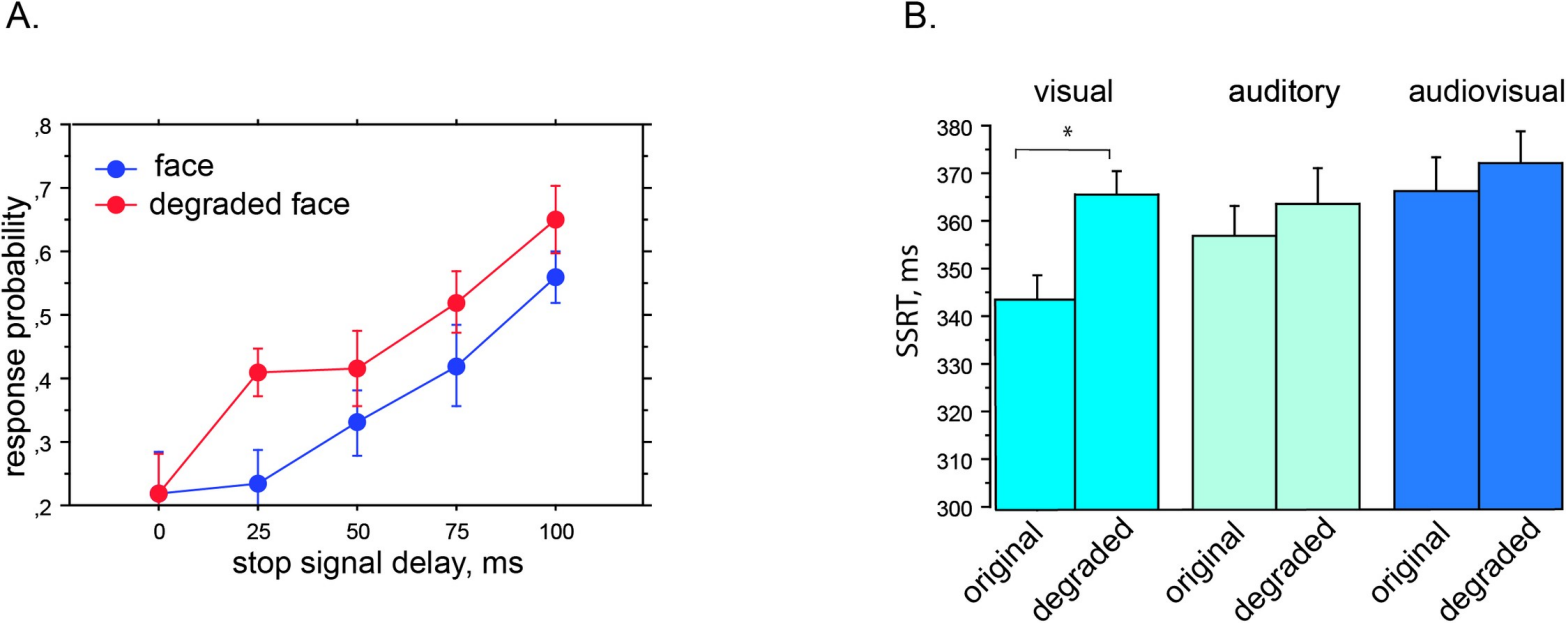
Effects of semantic load on response probability and stop–signal reaction time. *Note*. Panel A shows that response probability was lower for the original faces used as stop signals than for the degraded faces. This means that the original faces were more efficient in inhibiting action. Panel B indicates the effects of stop–signal modality and degradation on SSRTs. The effect of degradation on SSRTs was significant in the visual modality, as SSRTs were shorter for the original faces. * *p* < .05.

We then compared the original and degraded stop signals within each sensory modality. Using the paired Wilcoxon test for response probabilities, we showed that the original faces were a better stop signal than their degraded counterparts in the visual modality (*p* = .0002, *d* = .43, z = –3.72) (Fig 2A). This difference between the two did not depend on the SOA (Kruskal Wallis test, *p* = .08), as the probability of responding was significantly higher for the degraded faces regardless of duration (Fig 2A). There was no significant difference in response probabilities between original voices and degraded voices in the auditory modality (paired Wilcoxon test; *p* = .48, z = –0.71). Similarly, there was no significant difference in response probabilities between the original and degraded stop signals in the audiovisual modality (paired Wilcoxon test; *p* = .19, z = –1.31), meaning that inhibition capacity did not depend on the semantic content of the audiovisual stop signal.

#### Stop–signal reaction times

The ANOVA with the condition factor, *F*(5, 294) = 2.6, *p* = .026 (Fig 2B), revealed that there was only a significant difference in SSRT between the original and degraded versions of the stop signals in the visual modality (post hoc Fisher test; *p* = .013, *d* = .62). In the two–way ANOVA with the modality (visual, auditory, audiovisual) and semantic content (original, degraded) factors, semantic content had a significant effect, *F*(1, 294) = 5.2, *p* = .024, but the effect of modality only tended toward significance (*p* = .06), *F*(2, 294) = 2.8. The interaction between modality and semantic content was not significant, *F*(2, 294) = 1.1, *p* = .35. As indicated above, the modality effect was significant in the one–way ANOVA. A post hoc Fisher test after the two–way ANOVA indicated a significant difference between the audiovisual and visual modalities (*p* = .02), confirming the above–described result in the one–way ANOVA.

## Discussion

Our results indicate that, contrary to our initial hypothesis, a multisensory stop signal is much less efficient than a unimodal one in stopping an action. Response probabilities were higher for audiovisual stop signals than for unimodal ones. We found a similar contradiction of our initial hypothesis for the SSRTs calculated on the basis of the race model, as SSRTs were also longer for audiovisual stop signals. Further, semantic load only had a significant effect for visual stop signals, where the original faces were better stop signals than the degraded ones. No such effect of semantic load was observed for the audiovisual stop signals.

The most important result concerned the increase in response probabilities for audiovisual stop signals, compared with unimodal ones. We had hypothesized that multisensory facilitation (i.e., shorter RTs for audiovisual stimuli) would similarly lead to greater inhibition of motor responses, but results showed that audiovisual stop signals failed to provide a high inhibition level. On the contrary, the highest inhibition level was observed for unimodal stop signals, with lower response probabilities than those for the audiovisual modality. These results disproved our initial hypothesis but were in line with previous studies demonstrating poorer performances with multisensory signals in tasks involving executive control of action. For instance, in a go/no–go experiment, the rate of false alarms in no–go trials was higher in the visuotactile condition than in the visual one [36]. It is impossible to directly compare RTs for classic paradigms and stop–signal ones. However, SSRTs can be estimated using the independent race model for stop–signal tasks [13]. Our observation of lower inhibition levels for multisensory signals was corroborated by the SSRT analysis, as SSRTs were longer for bimodal stimulation, whereas in classic paradigms, RTs for bimodal stimulation are shorter.

To understand the *audiovisual impairment* demonstrated by our results, we can note that in most of the usual detection tasks showing a multisensory facilitation effect, the participants are cognitively free, ready to perform the task, and attentive to the upcoming stimulus. In contrast to this optimum situation, our participants were engaged in the main task when the stop signal occurred. This infrequent stimulation created a conflict in the monitoring of the main task, in which the participants had to stop what they were doing [37, 38]. In this particular situation of mental workload, we suggest that the additional sensory modality used for the bimodal stop signal increases the level of the conflict and, consequently, the task’s cognitive demand, leading to decreased efficiency in stopping the action. Instead of being processed as a unified multisensory object, as is proposed for audiovisual speech [39], the face–voice stop stimulus is processed as multiple concurrent stop signals, which slows down decision–making. However, as we clearly showed that semantic content is important (see Discussion below), the multisensory interaction in an inhibition task may depend on the type and combination of sensory modalities [36].

At the cortical level, audiovisual integration is known to modulate brain activity in multiple cortical sites, including low–and high–level networks [40]. The multisensory integration that takes place in the early stages of sensory processing is subsequently processed in motor–related cortical or subcortical areas and can enhance response outcomes [20, 21]. However, during a decisional conflict, multisensory integration modulates brain activity at higher cortical levels by increasing the cognitive effort needed to modify the response. Brain activity related to the less efficient response in the audiovisual modality has been associated with an increase in the P300 event–related potential, compared with activity related to more efficient responses in unimodal modalities [41]. This has been interpreted as reflecting the greater cognitive effort of simultaneously processing multiple inputs from different modalities. Thus, we may have observed higher response probabilities and longer SSRTs in the audiovisual modality because of heightened task demand. Participants had to monitor the conflict generated by the initiation of the response to the main task and the occurrence of an unexpected stop signal (which occurred in a minority of the trials). In this context, the concurrent information added by the bimodality of the signal may have impaired the simultaneous execution of central operations (i.e., responding to the main task stimulus, monitoring the conflict with the stop signal, inhibiting the response). Modality pairing may compete with the task demand and constitute a limitation in the form of a central bottleneck in the context of multitasking [42]. Loss of executive control in a decisional situation has been linked to perseveration errors in human operators [43]. There is also now evidence that unexpected salient stimuli can fail to reach awareness when individuals are engaged in cognitively demanding situations [44]. To sum up, when individuals are engaged in high–level conflict (e.g., response revision), multisensory signal presentation generates a high cognitive load. This increase in the supplementary task demand may impair action inhibition mechanisms.

In contrast to the inhibitory ineptness of the audiovisual stop signal in our study, the facial stop signal turned out to most efficiently inhibit action, reflected in the lower response probability and shorter SSRT. In the stop–signal paradigm, auditory signals are classically found to enhance both the speed and efficiency of response inhibition more than visual signals do [16, 45]. In our experiment, the facial stop signals in the visual modality led to better inhibition than the signals in the auditory and audiovisual modalities. This enhanced inhibition appears to have been face–specific, as it disappeared for the degraded faces with preserved spatial frequencies. *Face–specificity* means that inhibition is not merely mechanically triggered by a visual signal but that it is sensitive to the semantic content, which needs to be behaviorally meaningful to stop action. To the best of our knowledge, the face effect in visual stop–signal tasks had not previously been investigated, although the visual salience of the stop signal was already known to affect movement suppression, leading to shorter SSRTs and lower response probabilities [46, 47]. Faces constitute particularly salient stimuli, which we can detect and recognize significantly faster than nonfacial stimuli [48]. Moreover, a study using the anti–saccade paradigm showed that people have difficulty avoiding saccading to and fixating facial stimuli [49]. Thus, the salience of faces as stop signals renders inhibition faster and more accurate than with auditory stop signals. However, in our study, the visual face modality lost its efficacy to trigger the response inhibition process when it was combined with the auditory modality. The face facilitation seems not, therefore, to have been sufficient to offset the cost incurred by the increased task demand when concurrent multimodal information was added.

According to the standard horse–race model [12], the response initiation process (triggered by the main task stimulus) competes versus the response inhibition process (triggered by the stop signal). Thus, the result of the race determines whether the response is successfully canceled or not. The two processes are presumed to be stochastically independent (i.e., the finishing time of the response initiation process is independent of the finishing time of the response inhibition process in a given trial). However, the race model does not require functional independence between the two processes. A *functional* dependence would occur, providing a common factor was in capacity to influence both the duration of the response initiation and inhibition processes. Although this functional independence is still under debate [50], our results do not argue in favor of a functional linkage between the two processes. The multisensory content of a stimulation, known to facilitate the response to a go stimulus (see Introduction), appeared to damage the response to a stop signal. This impairment can probably be attributed to the inhibitory characteristic of the stop signal, rather than its unexpectedness. For instance, rare audiovisual stimuli were easier to detect than visual ones in an oddball task [51, 52]. This argues in favor of an audiovisual impairment in the perception of the stop signal, owing to its contradiction with the initiated main task response. One future avenue worth pursuing would be to test main task stimuli in different modalities (visual, auditory, audiovisual) to look for the possible interaction between the modality of the main task and that of the stop signal. It would also be worthwhile exploring the brain responses evoked by different modalities of the primary stimulus and stop signal within the same task to gain a better understanding of the multisensory facilitation and multisensory impairment processing stages.

Furthermore, individuals with attention deficit hyperactivity disorder exhibit both poorer inhibition of action [53, 54] and abnormal audiovisual integration, compared with neurotypical controls [55, 56]. Their inhibitory performances with visual stop signals could thus shed light on the interaction between cognitive control and modality pairing. The audiovisual impairment observed in the stop paradigm also suggests that redundant sensory information increases cognitive load and negatively impacts decision–making, a result that may apply to irrational actions or strategies observed among the operators of critical systems (e.g., aircraft pilots, automobile drivers) when confronted with multiple warning signals [43].

## Conclusions

Multisensory integration has been the subject of considerable research but has not yet been investigated in a situation calling for the executive control of thought and action. The present experiment makes it clear that modality pairing can have a dramatic effect on inhibitory control capabilities. Whereas visual (faces) stop signals were found to enhance inhibition, audiovisual stop signals led to higher response probabilities and longer SSRTs. We hypothesize that when people are engaged in an executive task, multimodal signals are not processed as a simple or unified object but as multiple sources of information. Concurrent information resulting from modality pairing in such a decisional situation may increase task demand and generate cognitive load, thereby impairing behavioral performance. However, an alternative explanation could be that subjects cannot disengage from the main active visual task’s attentional process. The specific for multisensory integration top–down attentional control mechanism [23] could be too late to engage a complex fronto–parietal network that would allow stopping the action. This delay could lead to momentary multisensory inattentional phenomena that result in perseveration behavior [43]. To the best of our knowledge, this is the first demonstration of an audiovisual impairment that contradicts the theoretical models (e.g., the co–activation model), which describe multisensory integration as a facilitator in the domain of perception and action.

## Supporting information

S1 File Theoretical inhibition function and response probabilities. (PDF)
