# Supplementary material for: When two is worse than one: The deleterious impact of multisensory stimulation on response inhibition
Source: PLoS One. 2021 May 20;16(5):e0251739. doi: 10.1371/journal.pone.0251739 (PMC8136741; doi:10.1371/journal.pone.0251739)
Supplement: S1 File — (PDF) [file pone.0251739.s001.pdf]

Theoretical inhibition function and response probabilities

To further confirm the validity of the SSRT computation, we verified whether our results corresponded to the theoretical inhibition function in the independent race model for stop–signal tasks of Boucher et al. [13]. Concerning how the model predicts the percentage of response, as described by Boucher et al., the amount of inhibition depends on the instantaneous activation levels (a “go” or a “stop”), causing a unit with a low activation to have a small inhibitory effect on the other unit. Together, these parameters specify the increment of activation on each time step toward a response threshold. The equations used by Boucher et al. are based on the leaky, competing accumulator model, which provides for the building up of different levels of activation and response probabilities. Thus, no information from the experimental results is required.

For the interested readers, we provide the differential equations of Boucher et al.:

$$da_{go}(t) = \frac{dt}{\tau}\left[\mu_{go} - k \cdot a_{go}(t) - \beta_{stop} \cdot a_{stop}(t)\right] + \sqrt{\frac{dt}{\tau}}\xi_{go} \; ; \qquad (1)$$

$$da_{stop}(t) = \frac{dt}{\tau}\left[\mu_{stop} - k \cdot a_{stop}(t) - \beta_{go} \cdot a_{go}(t)\right] + \sqrt{\frac{dt}{\tau}}\xi_{stop} \; ; \qquad (2)$$

These equations specify the change in unit activation (da_{go} and da_{stop}) within a time step dt (note that dt/τ was set equal to 1). The mean growth rates of the "go" and "stop" units are given by the go and stop parameters, respectively. ξ is a Gaussian noise term with a mean of zero and a variance equal to that of "go" or "stop". The leakage parameter, k, prevents the activation from increasing without bound, β is put to zero for independent race models.

When we applied Boucher et al. independent race model for stop–signal tasks to our SOA values, we obtained the theoretical inhibition function (Fig. 1S in this document), which

we compared with the mean response probabilities calculated for each modality in our study. The input provided to the model consists of stop–signal delays used in the experimental paradigm and the number of trials for each stop–signal delay. No information about the results of the experiment is provided to the model. We used the default parameters of the model defined in the toolbox of Boucher et al. (http://www.psy.vanderbilt.edu/faculty/palmeri/psyrev07_model/). Spearman correlations between the theoretical and experimental values were very high for each modality (*rho* = 1, $p = .017$). Regressions were significant at $p = .019$ for the auditory modality, at $p = .007$ for the visual modality and at $p = .004$ for the audiovisual modality (Fig. 1S in this document). Thus, our data corresponded rather closely to the theoretical inhibition function, allowing us to compute SSRTs based on the model.

Figure 1S ***Comparison of the race model inhibition function with response probability***

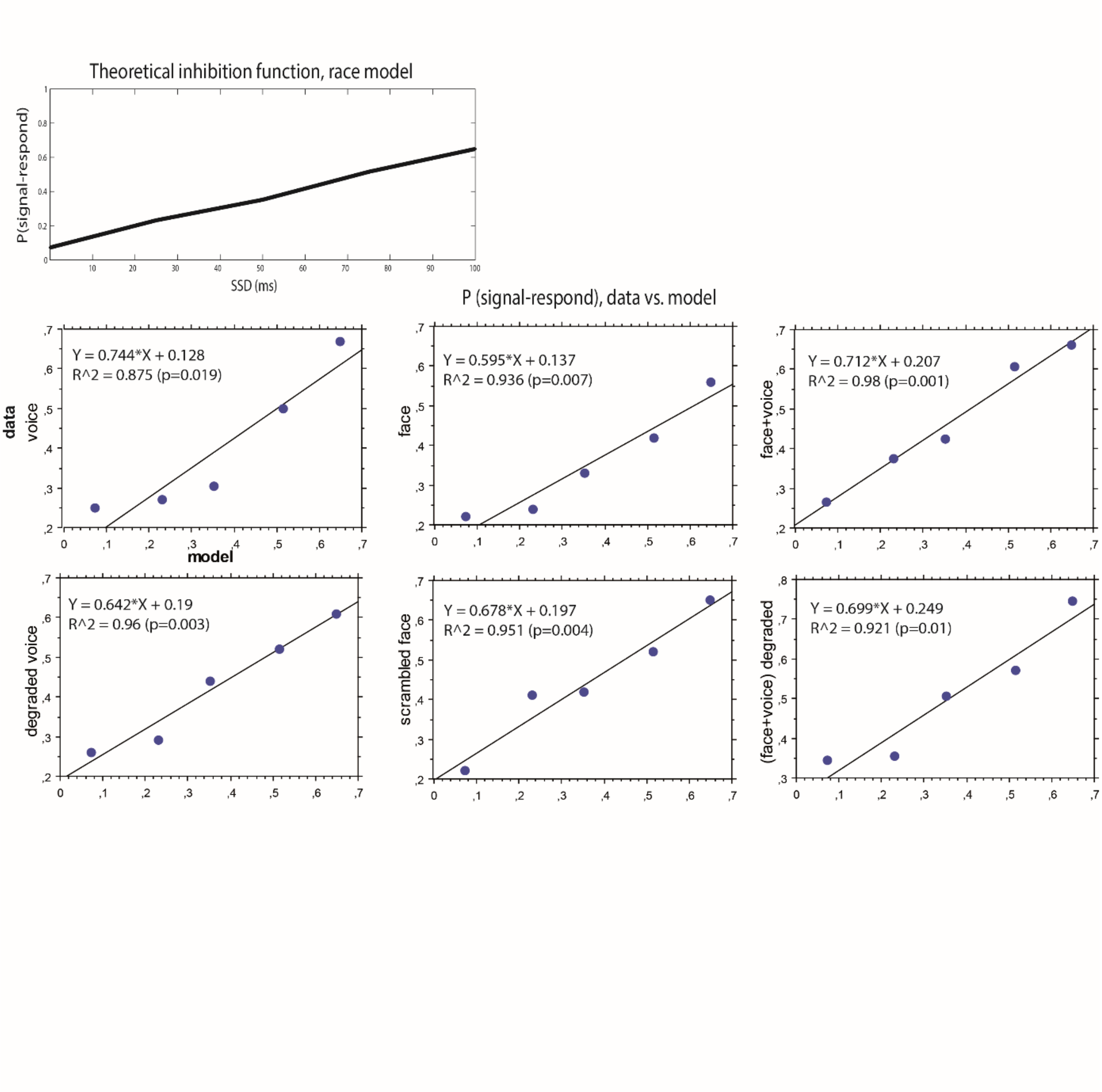

Note. The theoretical inhibition function based on the stop–signal delays in our study was calculated using the independent race model (Boucher et al., 2007). Correlations were then calculated between the response probability originating from the theoretical function and the real data for each type of stop signal, as illustrated in regression graphs.
